# ATR-FTIR Spectroscopy of Saliva and Machine Learning as a Screening Test for Sjögren Disease

**DOI:** 10.1021/acs.analchem.5c04238

**Published:** 2025-11-18

**Authors:** Jhonatan Contreras, Bhavik Vyas, Oleg Ryabchykov, Melinda Larsen, Neil Gildener-Leapman, Thomas Bocklitz, Jürgen Popp, Igor K. Lednev

**Affiliations:** † Photonic Data Science Department, 27716 Leibniz Institute of Photonic Technology (IPHT), Albert-Einstein-Straße 9, Jena 07745, Germany; ‡ Institute of Physical Chemistry & Abbe Center of Photonics (ACP), Friedrich Schiller University Jena, Helmholtzweg 4, Jena 07743, Germany; § Department of Chemistry and Center for Biophotonic Technology and Artificial Intelligence (CeBAI), 1084 University at Albany, SUNY, Albany, New York 12222, United States; ∥ Division of Otolaryngology Head and Neck Surgery, 1092 Albany Medical College, Albany, New York 12208, United States; ⊥ Department of Biological Sciences and the RNA Institute, 1084 University at Albany, SUNY, Albany, New York 12222, United States

## Abstract

Sjögren’s disease is often an underdiagnosed autoimmune condition that primarily affects the exocrine glands, resulting in symptoms such as dry eyes and dry mouth. Diagnostic challenges stem from nonspecific symptoms, the absence of definitive biomarkers, and the invasiveness and cost associated with current clinical tests. This study investigates the application of attenuated total reflectance Fourier-transform infrared spectroscopy (ATR-FTIR), in conjunction with a neural network and Monte Carlo dropout-based uncertainty estimation to distinguish patients with Sjögren’s disease from healthy individuals. Uncertainty estimates were used to identify ambiguous spectra and refine training data, improving predictive performance and enabling confidence-aware interpretation. To minimize bias related to sex-based prevalence, model training was limited to female participants, among whom the disease is more common. To guarantee independent validation, a stratified cross-validation strategy was used. In each fold, the network is initialized with random weights, trained exclusively on the training subset, and evaluated on the corresponding unseen data. The refined (uncertainty-aware) model achieved 75% accuracy and 73% sensitivity at the individual spectrum level, and 79% accuracy and 76% sensitivity at the patient level. These findings highlight the potential of an uncertainty-aware, noninvasive, machine learning–based method as a reliable tool for the early diagnosis of Sjögren’s Disease.

## Graphical abstract

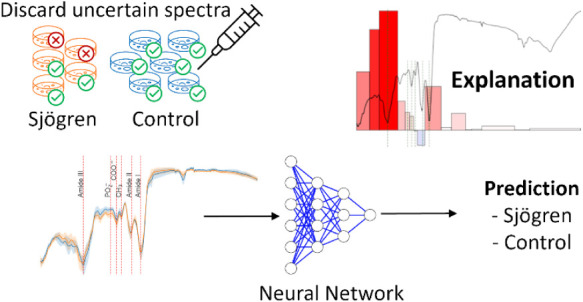

## 1 Introduction

Sjögren’s Disease (SjD) is a systemic autoimmune disorder characterized by lymphocytic infiltration, causing progressive dysfunction of the exocrine glands, primarily the lacrimal and salivary glands. This leads to the classic symptoms of xerostomia (dry mouth) and keratoconjunctivitis sicca (dry eyes).^1^ In the United States, it is estimated that between 1 and 4 million individuals are affected by this condition, with a significantly higher prevalence among women, particularly those over the age of 40 years. Sjögren’s disease is often underdiagnosed due to its diverse range of symptoms and overlap with other autoimmune diseases.^2^

The disease can be classified into two categories: primary and secondary Sjögren’s disease. Primary Sjögren’s disease manifests as an isolated autoimmune disorder without accompanying conditions. In contrast, secondary Sjögren’s disease occurs in conjunction with other systemic autoimmune diseases, such as rheumatoid arthritis or systemic lupus erythematosus. In addition to the characteristic symptoms of xerostomia (dry mouth) and dry eyes, patients diagnosed with Sjögren’s disease may also confront systemic manifestations, which include arthralgia (joint pain), pronounced fatigue, and potential involvement of the pulmonary, renal, and nervous systems. These multisystemic effects underscore the complexity and variability inherent in Sjögren’s disease, necessitating a holistic approach to diagnosis and management.^3^

The prevalence of Sjögren’s Disease is significantly higher in females compared to males, irrespective of race and geographic location, with an estimated female-to-male ratio of 9:1^2^. While the exact etiology of Sjögren’s Disease remains unknown, it is believed to arise from a genetic predisposition and inaccuracies in environmental and viral triggers. Genetic predisposition, particularly certain variants of HLA genes, has been linked to a higher likelihood of developing Sjögren’s Disease.^4^ Additionally, it has been suggested that environmental factors—such as viral infections, especially the Epstein–Barr virus—could trigger an autoimmune response. The role of hormonal influences in its onset is also considered a possibility, particularly given that the case histories have been predominantly female.^5^

The classification of Sjögren’s disease poses considerable complexity and controversy. The American College of Rheumatology (ACR) in conjunction with the European League Against Rheumatism (EULAR) has recently established a consensus on a set of criteria, which were last revised in 2016.^6^ The diagnosis of Sjögren’s disease involves a comprehensive clinical assessment, serological tests, salivary gland biopsies, and imaging techniques, necessitating a score of 4 out of 5 tests.^7^ Typically, the diagnosis relies on the presence of specific characteristic autoantibodies, particularly against SSA/Ro and SSB/La; however, numerous patients may test negative for all these biomarkers. Salivary gland biopsies are regarded as the gold standard for diagnosis, as they identify focal lymphocytic sialadenitis and the presence of germinal centers.^8^ Nonetheless, even this invasive procedure does not always yield unequivocal results. Furthermore, patients are referred to an ophthalmologist to evaluate their lacrimal production through Schirmer’s test and to examine the integrity of the epithelial layers of the cornea and conjunctiva via ocular staining.^9^ Currently, no singular evidence-based standardized screening test exists that can effectively diagnose individuals who present with complaints of dry mucous membranes. Due to the complexity surrounding diagnosis and the variability of patient symptoms, there exists a persistent underdiagnosis of the disease,^10^ thereby restricting the capacity to initiate therapy promptly or to accurately recruit patients for clinical trials. As a result, diagnosis is often significantly delayed, hindering early treatment and heightening the risk of irreversible organ damage.

Current diagnostic pathways for Sjögren’s disease are fragmented, invasive, and often inconclusive, leaving many patients undiagnosed for years while irreversible glandular damage progresses. There is an urgent need for a single, objective, noninvasive test that can overcome the limitations of serology, biopsy, and symptom-based criteria. By applying ATR-FTIR spectroscopy with machine learning to saliva, this project addresses that critical gap and lays the foundation for a rapid, reproducible, and scalable screening tool if not diagnostic tool with clear potential for FDA translation and clinical adoption.

Since most diagnostic tools have limitations, it is essential to develop new biomarkers for diagnosing Sjögren’s disease more accurately and noninvasively. Recent studies have concentrated on disease activity and progression, possibly indicated by molecular signatures found in saliva, tears, and blood. These biomarkers could improve diagnostic precision and facilitate early detection, laying the groundwork for personalized treatment strategies.^1,8,9^

Fourier Transform Infrared (FTIR) spectroscopy has emerged as a highly effective diagnostic tool, providing noninvasive, detailed insights into molecular structures. This technique facilitates both qualitative and quantitative analyses by assessing how molecules absorb infrared radiation, making it suitable for various sample types, including solids, liquids, and biofluids. FTIR has been successfully applied in diagnosing conditions like cancer, diabetes, and neurodegenerative diseases by identifying specific molecular alterations in patient samples.^11,12^ Research has shown its ability to distinguish between healthy and diseased individuals through the analysis of changes in biofluids, such as serum and saliva.^13^

Vyas et al. successfully distinguished patients diagnosed with Sjögren’s disease from healthy control subjects and from individuals receiving radiation therapy for head and neck tumors. This differentiation was accomplished through the application of Raman hyperspectroscopy of saliva, supplemented by advanced machine learning algorithms.^14^ This significant breakthrough underscores the potential of noninvasive salivary diagnostics for clinical applications.

Building on this foundational premise, the present study explores the application of Attenuated Total Reflectance-Fourier Transform Infrared (ATR-FTIR) spectroscopy for identifying and distinguishing Sjögren’s disease patients from healthy controls. While previous research primarily focused on Raman spectroscopy, this study expands the scope of spectroscopic methodologies, emphasizing their complementary roles in enhancing diagnostic precision. FTIR spectroscopy has demonstrated substantial sensitivity in detecting saliva composition changes associated with neurodegenerative diseases such as Alzheimer’s and Parkinson’s, underscoring its potential as a powerful diagnostic tool.^15–17^

The incorporation of ATR-FTIR spectroscopy into the diagnostic framework for Sjögren’s Disease marks a significant advancement in noninvasive disease detection. By leveraging its ability to detect biochemical changes in saliva, this study aims to identify distinct spectral markers for Sjögren’s Disease. The results could improve early diagnosis and disease monitoring, ultimately broadening the role of spectroscopic techniques in precision medicine and fostering the development of accessible, noninvasive diagnostic tools.

## 2 Materials and Methods

### 2.1 Saliva Samples

Saliva samples were collected from 47 donors (one sample per donor: 23 controls, 24 with Sjögren’s Disease) at Albany College of Medicine, following approval by the Institutional Review Board (IRB). Samples were stored at −80 °C until further analysis. Age demographics for the donors are provided in Figure 1.

**Figure 1:**
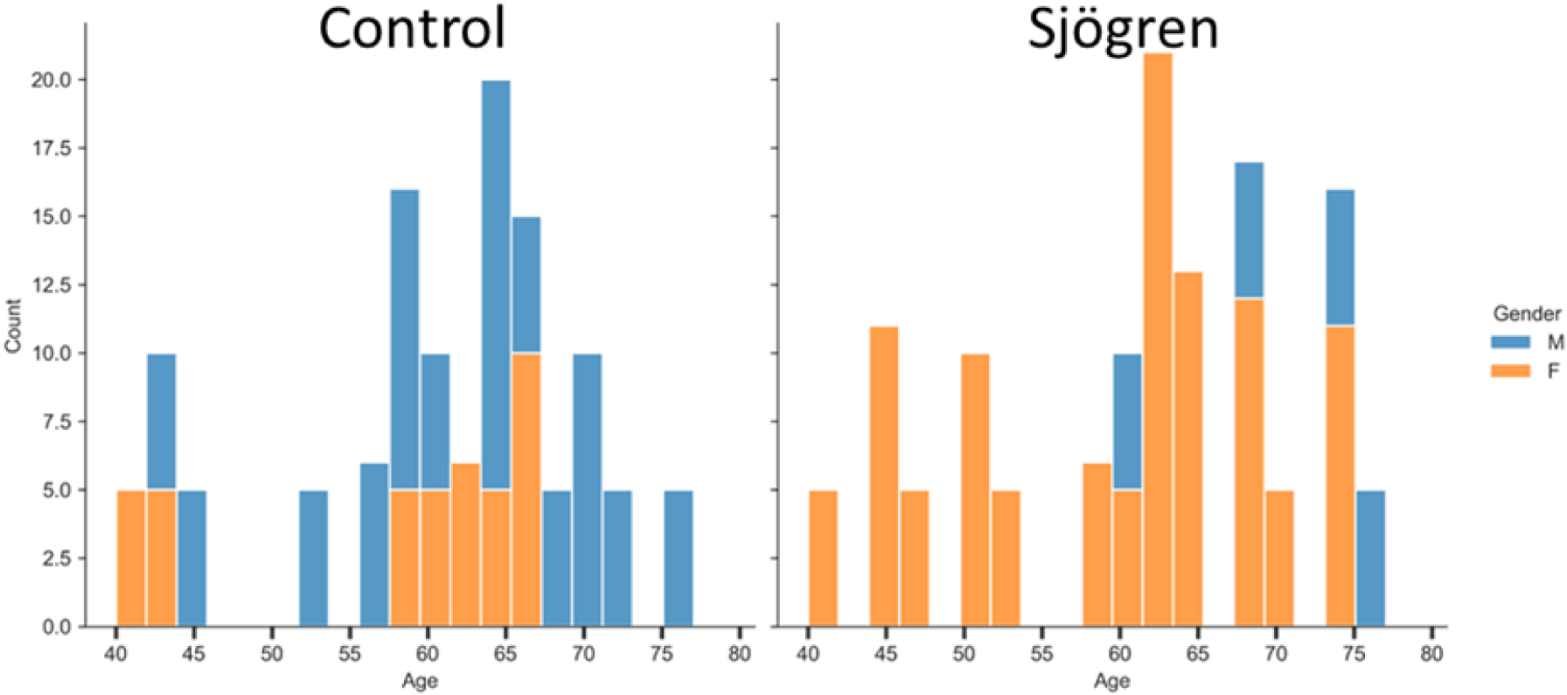
Age and gender distribution of all collected donor samples in the control and Sjögren’s disease groups. Each bar shows the number of male (blue) and female (orange) spectra in each age bin. Although both genders were included in the initial exploratory analysis, only female participants were used in the primary modeling in order to reduce demographic bias and make more meaningful biological comparisons. Within the female cohort, the age distributions between groups are more balanced, with most donors falling within the 57–70 age range.

Participants were instructed to avoid eating, drinking, chewing gum, or smoking for at least 30 min prior to sample collection. Patients in the Sjögren’s disease group were clinically diagnosed by rheumatologists according to the American College of Rheumatology/European League Against Rheumatism (ACR/EULAR) classification criteria. Eligible participants were under active treatment, reported xerostomia, demonstrated evidence of multiorgan involvement, and tested positive for Anti-SSA/Anti-SSB antibodies or at least one of the Early Sjögren’s disease-associated antibodies. Donors in the control group were selected from individuals without symptoms of oral dryness, no identifiable oral health concerns, and no prior history of oral cancer.

Saliva samples were kept frozen at −80 °C until they were ready for analysis. After thawing, the samples were centrifuged at 20,000 rpm for 5 min, and the supernatant was collected for ATR-FTIR spectral analysis. A 20 μL aliquot of the supernatant was placed on a glass slide covered with aluminum foil and air-dried prior to transferring it to the ATR crystal for measurement. This method of drying the sample on the aluminum foil before placement in the ATR crystal enhanced analysis efficiency by minimizing measurement time and boosting throughput.^18^

### 2.2 Gender Stratification and Dataset Refinement

Sjögren’s Disease exhibits a marked gender disparity, with approximately 90% of patients being female. In our data set, only four out of 24 patients with Sjögren’s disease were male, resulting in a pronounced gender imbalance (Figure 1). We performed an exploratory modeling on the full data set, including both male and female participants, to evaluate the overall feasibility of spectral classification. However, our primary modeling analyses used only female participants from the Sjögren and control groups to reduce demographic bias and make more biologically meaningful comparisons.

### 2.3 Instrumentation

We analyzed the samples using a PerkinElmer Spectrum 100 FT-IR spectrometer (PerkinElmer, Inc., Waltham, MA), equipped with a diamond/ZnSe ATR crystal. The analysis was performed with Spectrum software version 6.0.2.0025 (PerkinElmer, Inc., Waltham, MA). The spectral range was set from 4000 to 650 cm^–1^, with a resolution of 4 cm^–1^. We collected five spectra, each consisting of five scans, from various locations of dry saliva stains to capture the sample’s heterogeneity. Between scans, we cleaned the crystal with ultrapure water and ethanol using an antidust wipe to ensure accurate readings. Additionally, we recorded an air spectrum for background reference prior to examining each sample. All spectra were recorded at room temperature, maintained between 22 and 23 °C.

### 2.4 Spectral Preprocessing

All spectral data were preprocessed using model-based Extended Multiplicative Signal Correction (EMSC)^19^ to ensure consistency (see Figure 3). A sixth-degree polynomial was applied within the EMSC framework to correct for baseline drift and multiplicative effects, common sources of variability in ATR-FTIR spectroscopy.^20^ After preprocessing, the spectra were cropped to the region between 650 and 3700 cm^–1^, where most of the relevant molecular absorption bands are located. This spectral window includes vibrational modes associated with proteins, lipids, nucleic acids, and carbohydrates commonly altered in systemic diseases such as Sjögren’s disease.

### 2.5 Classic Chemometric Modeling

As a base approach, Principal Component Analysis (PCA)^21^ was employed to reduce the dimensionality and noise of the spectra while preserving most of the variance in the data set. The number of retained principal components (PCs) was selected based on cumulative explained variance and empirical testing.

The transformed data obtained from PCA served as input to a linear discriminant analysis (LDA) classifier.^22,23^ LDA is a supervised method that aims to maximize the separation between predefined classes, in this case, Sjögren’s disease and healthy controls, by projecting the data onto a linear discriminant space.

### 2.6 Neural Network-Based Classification

To overcome the limitations of linear classifiers and dimensionality selection, we implemented a neural network model optimized explicitly for spectral data: RamanNet.^24^ RamanNet is a domain-specific architecture designed to process 1D spectral inputs. It divides the input spectrum into overlapping windows, which are then passed through dense blocks that simulate the behavior of sparse convolutional networks. This windowed approach allows the model to focus on local spectral features while maintaining efficient parametrization.

A neural network was trained to classify spectra as originating from patients with Sjögren’s disease or healthy controls. Initial training revealed the presence of ambiguous or mislabeled spectra, particularly in the Sjögren’s group, which negatively impacted model generalization. These problematic spectra may be due to biological heterogeneity in clinical annotations.

A 7-fold stratified cross-validation strategy was implemented to assess the reliability of the classification. In each fold, a RamanNet model is initialized with random weights, trained for 200 epochs exclusively on the training subset, and evaluated on the corresponding unseen test data. Performance metrics such as sensitivity (true positive rate) and accuracy (overall correctness of predictions) were used to evaluate model effectiveness.

### 2.7 Dropout Regularization

Dropout is a regularization technique introduced to reduce overfitting in neural networks by randomly deactivating a subset of neurons during training.^25^ This forces the network to learn redundant representations, which improves generalization. Mathematically, it involves multiplying the inputs or hidden layer activations by a binary mask sampled from a Bernoulli distribution, effectively “dropping” units with a fixed probability.

During inference, the dropout is typically disabled, and the network uses the full set of weights, scaled to account for the units that were disabled during training. This technique has been shown to have effects similar to L2 regularization and is widely used in convolutional and fully connected networks.

### 2.8 Monte Carlo Dropout (MCD) for Uncertainty Estimation

Uncertainty estimation is critical in applications such as biomedical diagnostics, where model outputs must be accurate and trustworthy. Several approaches that quantify uncertainty in deep learning models exist. Among the most common are model ensembling, test time data augmentation, and bootstrapping-based methods.^26–28^ These techniques generate multiple predictions, either by training multiple models or by perturbing the input data, and then assess uncertainty based on the variability of these outputs.

While ensembling, training multiple independent models, and aggregating their predictions can provide robust uncertainty estimates, they come at a significant computational cost, especially in resource-constrained settings or when working with small data sets. Bootstrapping, on the other hand, assesses model stability by resampling the training data but does not provide sample-specific uncertainty at inference time and requires multiple training iterations. These limitations make such approaches less suitable for our context, which involves limited annotated spectroscopy data. Additionally, in our case, the goal of uncertainty estimation was not to capture the complete uncertainty profile of the model but rather to identify and retain the most confidently predicted samples from a subset of the data that may contain mislabeled or ambiguous entries. This selective approach allowed us to refine the training data set and improve model generalization by focusing on the most reliable examples.

To address these challenges, we adopted Monte Carlo Dropout (MCD), a method proposed by Gal and Ghahramani (2016),^29^ that extends dropout to the inference phase to approximate Bayesian uncertainty in deep neural networks. Unlike ensembling or bootstrapping, MCD requires no architectural changes and involves only a single trained model, making it computationally efficient and well-suited for our setting. During inference, MCD keeps dropout active and performs *T* stochastic forward passes through the model. For a given input *x*, this results in a distribution of predictions:

$$ŷ=\frac{1}{T}\overset{T}{\underset{t=1}{\sum }}f^{\left(t\right)}\left(x\right)$$

$$Var\left(y\right)=\frac{1}{T}\overset{T}{\underset{t=1}{\sum }}\left(f^{\left(t\right)}{\left(x\right)}^{2}\right)-{ŷ}^{2}$$

Here, *f* ^(*t*)^(*x*) represents the model’s prediction at the *t* ^*th*^ stochastic pass, ŷ represents the mean prediction of the model output, and the variance estimates the model uncertainty without requiring any training procedure or architecture changes. In our work, we use MCD to quantify uncertainty for each individual spectrum and to guide sample selection in a two-stage training strategy (see Classic Chemometric Modeling).

### 2.9 Uncertainty-Based Sample Selection via Monte Carlo Dropout

To enhance classification robustness and address potential label noise, particularly within the Sjögren’s spectra, we utilized Monte Carlo Dropout (MCD) during inference, which allows the estimation of prediction uncertainty.

A 7-fold stratified cross-validation strategy was employed. Each fold has two phases. In the first training phase, a RamanNet model is initialized with random weights and trained for 200 epochs exclusively on the training subset, which includes spectra from both healthy controls and Sjögren’s patients. After training, MCD was used to compute uncertainty scores for each spectrum within the training set. For each patient with Sjögren’s disease, spectra with high uncertainty scores were considered unreliable, likely due to ambiguous biochemical profiles and were excluded from the subsequent second phase training iteration. A minimum of 20% (one or two) spectra per patient was retained. For the control group, all spectra were kept for training in the second phase.

The rational for this is that if one assumes that the amount of biomarkers specific to Sjögren’s disease is relatively small, then not all locations on a dry sample stain might contain the biomarker or its local concentration might be small resulting in a small contribution to the corresponding spectrum. Such a spectrum might not be helpful for the training data set because it closely resembles that of a healthy control spectrum. However, all spectra from a healthy control sample should be healthy, so all of them are good for the training data set. In the second training phase, a new model is initialized with random weights and trained for 200 epochs using the entire set of control spectra and the low-uncertainty Sjögren’s group spectra. This reinitialization is necessary because retaining the weights of the previous model often leads the optimizer to remain trapped in a local minimum influenced by noisy samples from the first training phase. This selective learning strategy reduces the impact of noisy or overlapping samples, thereby improving model generalization and class separability. However, for the prediction of test folds, all spectra are considered, and majority voting is employed for donor-level decision making. Figure 2 illustrates this two-phase training workflow.

**Figure 2:**
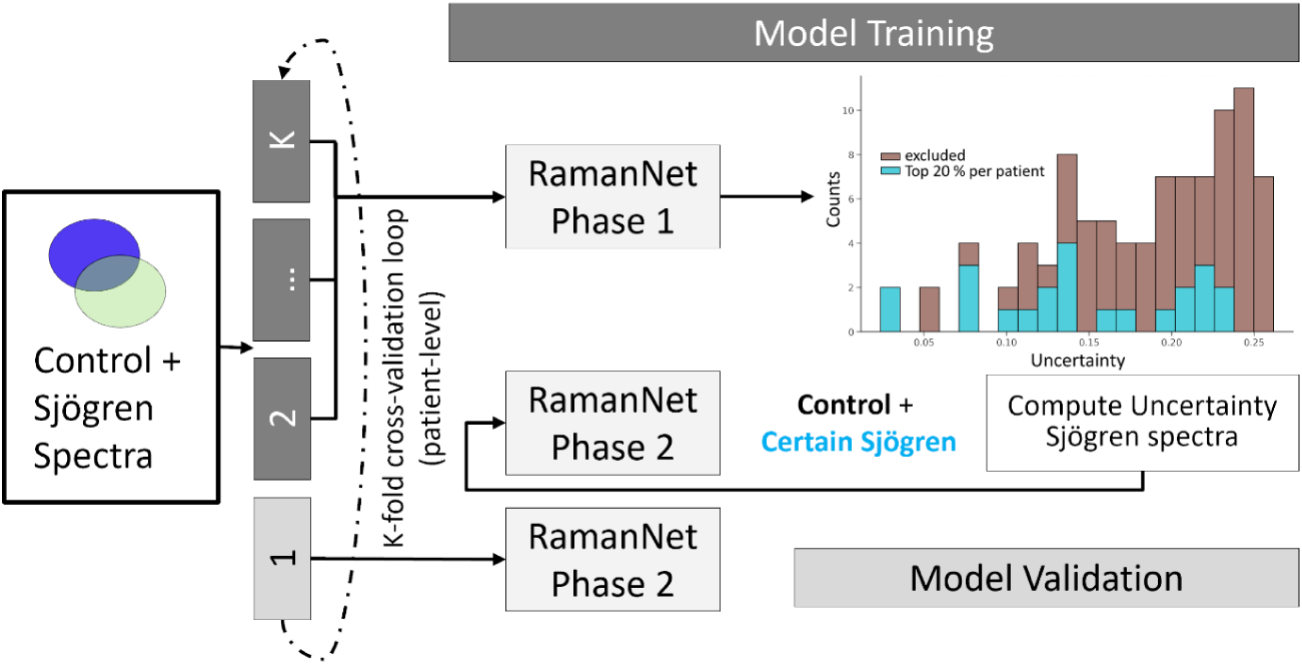
Two-phase training strategy using Monte Carlo Dropout (MCD) for uncertainty-based sample selection. The complete data set is fed into the neural network in the top panel, and uncertainty is estimated using the MCD method. In the second phase, all control spectra and at least 20% of spectra per patient (shown in cyan) are retained for training. In the bottom panel, the model is retrained using this refined data set, which includes all control samples and low-uncertainty Sjögren’s spectra, resulting in increased interclass variance, so the model learns most relevant features. In the model validation, all control spectra and all spectra from patients are kept to avoid data leakage and making a fair evaluation of spectral-level model performance possible.

## 3 Results and Discussion

### 3.1 ATR-FTIR Spectral Properties of Sjogren’s Disease and Control Saliva

ATR spectra were collected from 47 donors (23 controls, 24 with Sjögren’s Disease) and initially evaluated by visual inspection to identify any outliers. The original ATR-FTIR spectra were processed using model-based Extended Multiplicative Signal Correction (EMSC) to ensure consistency (see Figure 3). A sixth-degree polynomial was applied within the EMSC framework to correct for baseline drift and multiplicative effects, both of which are common sources of variability in ATR-FTIR measurements. We further normalized the spectra for baseline oscillations, scattering effects, and other instrumental irregularities, such as temperature fluctuations and environmental factors. This preprocessing step ensured that the resulting spectra reflected true biochemical signatures rather than artifacts.

**Figure 3:**
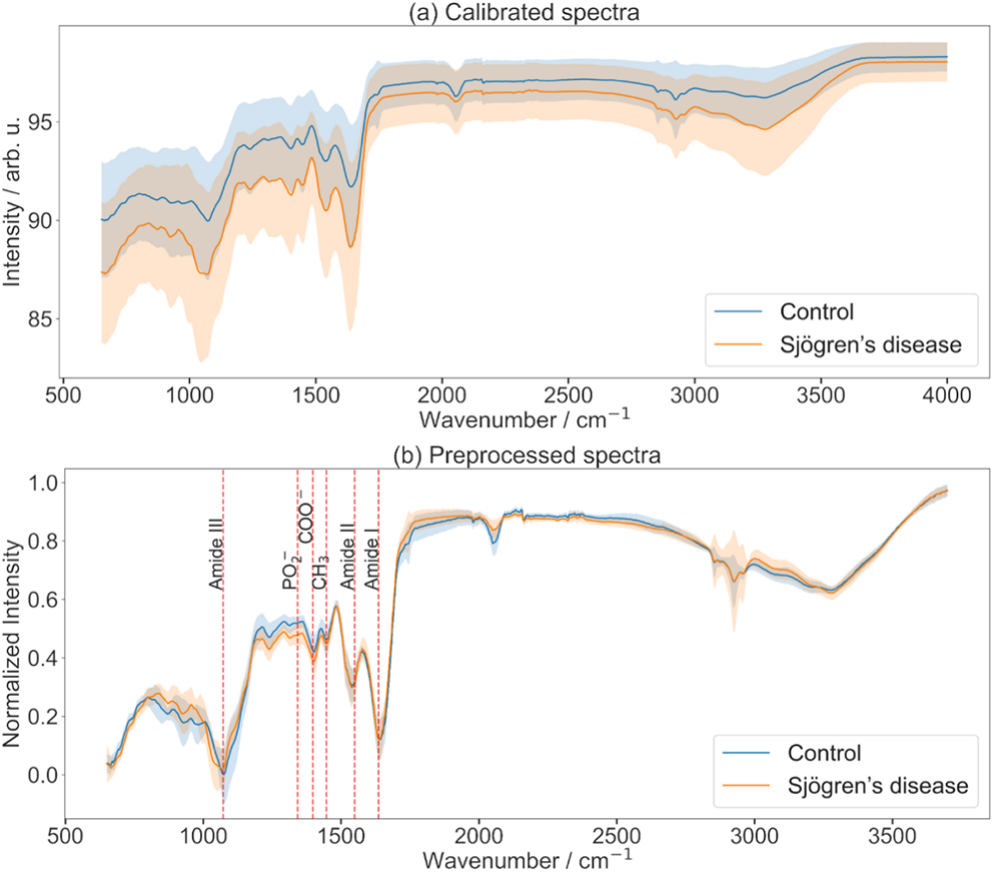
Average ATR-FTIR spectra of human saliva from control and Sjögren’s disease donors. (a) Raw, unprocessed average spectra for the control group (blue) and Sjögren’s disease group (orange). Shaded regions represent the ± 1 standard deviation within each class, indicating intersample variability. (b) Corresponding preprocessed average spectra of the same sample sets, with the same color scheme and standard deviation shading. Absorption bands highlighted according to Table 1.

After preprocessing, spectra were cropped to the 650–3700 cm^– 1^ region, where most molecular absorption bands are found. This region includes characteristic vibrational modes associated with proteins, lipids, nucleic acids, and carbohydrates that are commonly altered in systemic diseases such as Sjögren’s disease.

Characteristic band assignments of molecular vibrations in human saliva spectra can be found in Table 1. Spectral features observed in this study are consistent with those reported in the literature.^30^ For example, protein content is primarily associated with amide I and amide II bands at 1636 cm^– 1^ and 1549 cm^– 1^, respectively. Lipid contributions are evident at 1404 cm^– 1^. Saliva is a complex and dynamic biological fluid composed of DNA, RNA, proteins, and metabolites. The salivary proteome exhibits remarkable dynamism, comprising over 3,652 distinct proteins and 12,562 peptides.^31^ Notably, it shares approximately 51% of its proteins and 79% of plasma-derived peptides. Consequently, saliva may serve as a valuable medium for the screening and early detection of diseases, such as autoimmune disorders like Sjögren’s disease, which we have targeted in our research.

**Table 1:** Assignments of Absorption Bands in the Average Saliva ATR-FTIR Spectra Based on Literature.^30,32,33^

Peak (cm^–1^)	Proposed vibrational mode	Molecular source
1636	Amide I [ν (C = O), ν (C–N), δ (N–H)]	Protein
1549	Amide II [ν (N–H), ν (C–N)]	Protein
1447	CH_3_ asymmetric bending [δ_as_ (CH_3_)]	Protein (methyl groups)
1398	COO^–^ symmetric stretching [ν_s_ (COO^–^)]	Lipid (fatty acids)/Protein (amino acids)
1342	PO_2_^–^ Asymmetric Stretching	Nucleic acid (phosphodiester group)/Phospholipid/Phosphorylated protein
1072	Amide III (C–N), Symmetric stretching (O–H stretching) and bending (C–O) stretching and bending (C–OH)	Protein (e.g., collagen) Nucleic acid (RNA/DNA) Carbohydrates (glycogen, glucose)

### 3.2 Interpretation of SHAP Values for Sjögren’s Disease Classification

We employed a machine learning approach based on RamanNet, a model architecture optimized for spectral data analysis. Although neural networks like RamanNet have demonstrated strong predictive performance, they are often criticized for being opaque and complex to interpret^34^ due to their black-box nature.

To address this challenge and gain insight into the model’s reasoning, we applied explainable artificial intelligence (XAI) techniques. Specifically, we analyzed SHapley Additive exPlanations (SHAP)^35^ values derived from the trained model to identify which spectral regions are most influential in distinguishing Sjögren’s disease from control samples.

Figure 4 shows a SHAP summary plot for the Sjögren class. Each feature corresponds to a wavenumber range, called a spectral zone, and each point represents an individual spectrum. The horizontal position reflects the SHAP value, indicating whether a given feature pushes the model toward (positive values) or away from (negative values) the Sjögren prediction. The color represents the feature value: red for high spectral intensity and blue for low (high absorbance).

**Figure 4:**
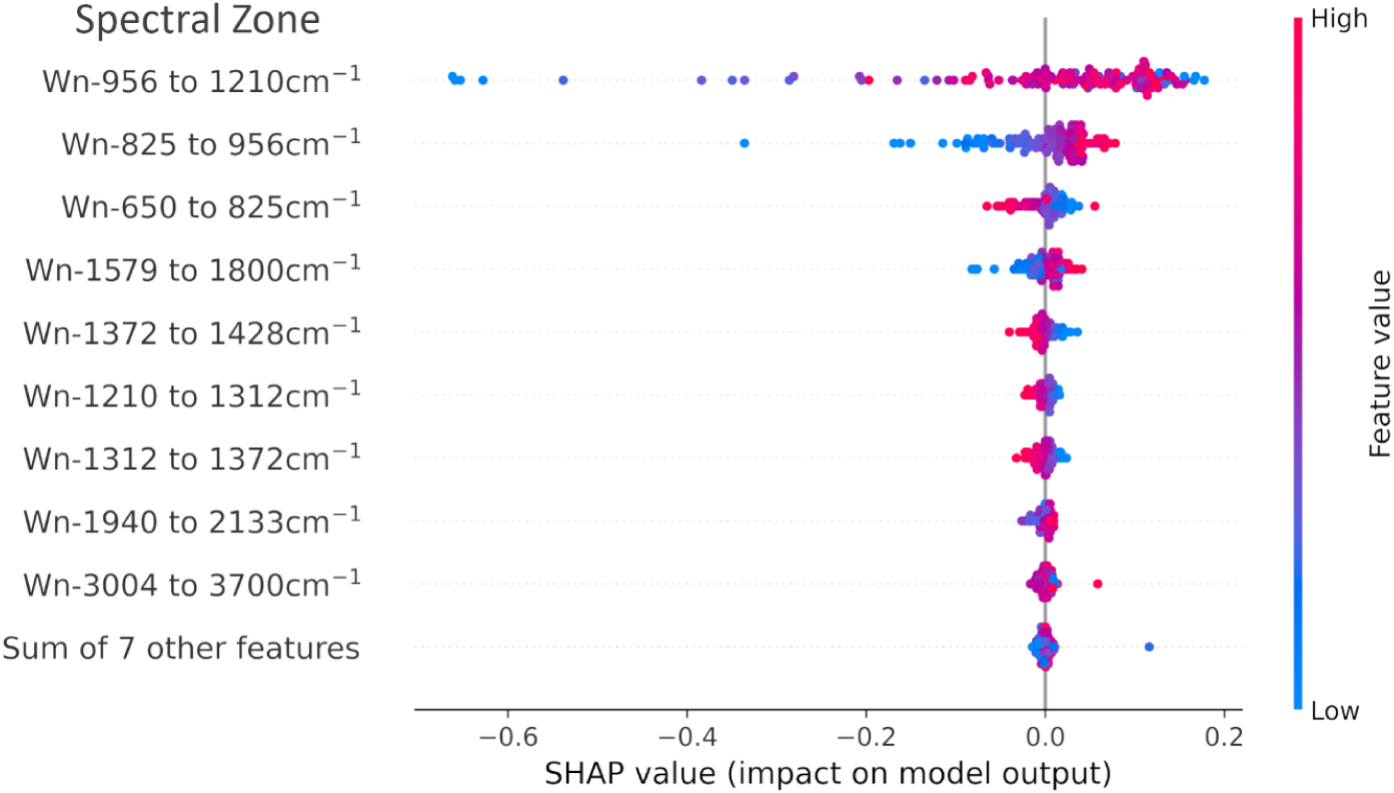
SHAP summary plot for the Sjögren’s disease class. Each point represents a spectrum, and each horizontal line corresponds to a spectral zone (wavenumber range). The horizontal position of each point indicates its SHAP value, which reflects how much that feature contributes to increasing or decreasing the model’s prediction of Sjögren’s disease. The color represents the normalized average spectral intensity within the zone (red = high; blue = low).

The most impactful regions were 956–1210 cm^–1^ and 825–956 cm^–1^, with high spectral intensities positively associated with Sjögren’s predictions. This region is associated with Amide III (C–N), symmetric stretching (O–H stretching), bending (C–O) stretching and bending (C–OH), and carbohydrates. This could reflect a decreased presence of nucleic acids and glycoproteins in the saliva of affected individuals.

The zones 825–956 cm^–1^and 1579–1800 cm^–1^ range, which include the Amide I peak, showed that high absorbance (blue points) in these regions reduces the model’s confidence in predicting Sjögren’s disease. This suggests a potential loss of protein structure, degradation, or altered protein content in affected individuals. While the zone 650–825 cm^–1^ showed that lower intensities (blue points), higher absorbance in these regions increased the model’s confidence in predicting Sjögren’s disease. To interpret the model’s decisions locally (at spectra level), we examined the zone-level SHAP at two resolutions. Figures 5 and S1 (Supporting Information) provide an example of zone-level SHAP attribution for a correctly classified Sjögren spectrum. The height of each bar represents the absolute magnitude of the SHAP value, and the color indicates its direction and intensity (red indicates a positive contribution, and blue indicates a negative contribution).

**Figure 5:**
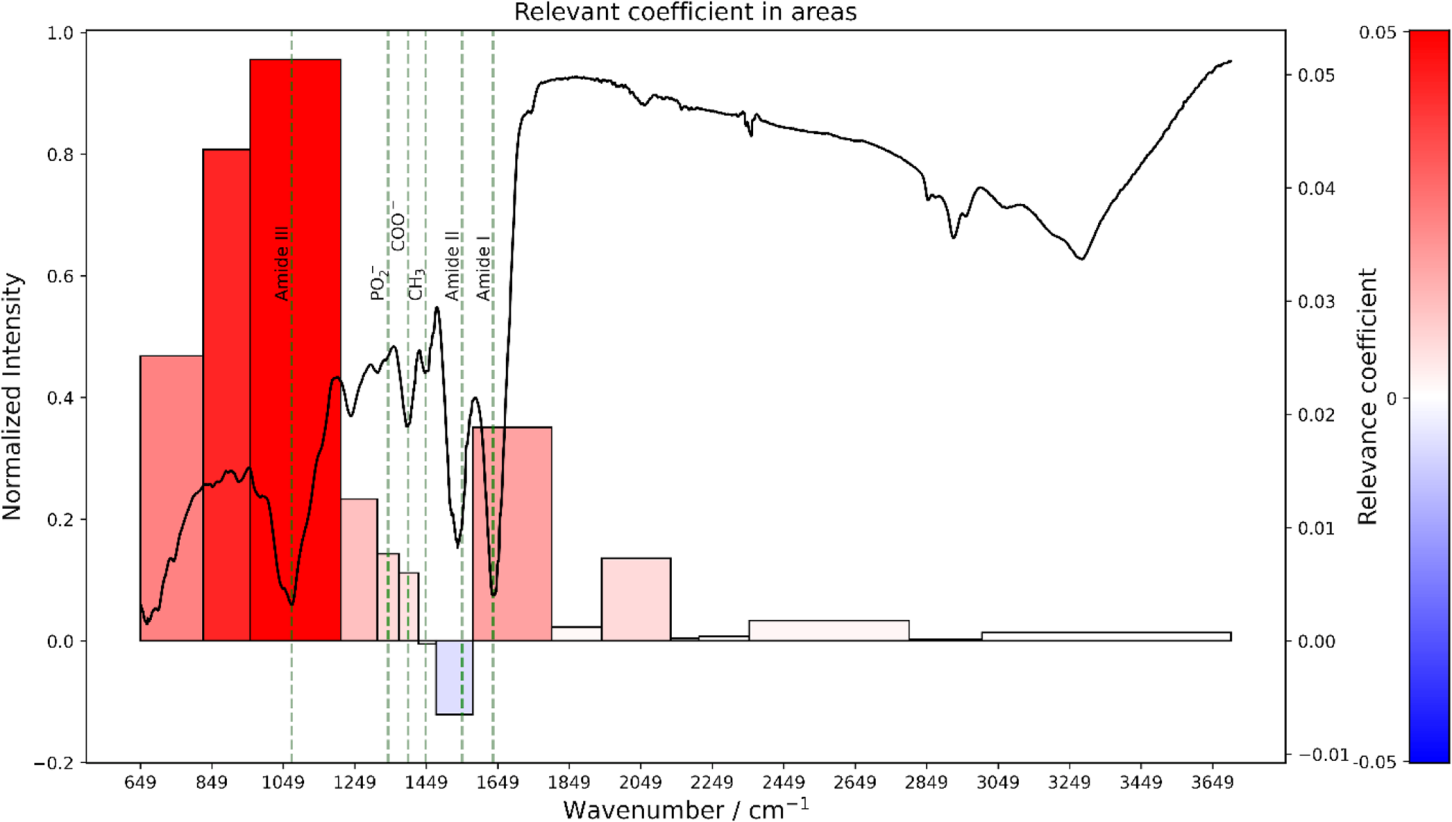
Zone-Level SHAP Attribution for an individual spectrum corresponding to Sjögren’s Disease. Bar height reflects the magnitude of each zone’s SHAP value, and color indicates whether the contribution to the Sjögren prediction was positive (red) or negative (blue). This case-specific explanation shows how the model attributes disease prediction to spectral features associated with the biochemical alterations.

The first resolution, shown in Figure 5, aggregates neighboring bands into broader features. The second resolution, shown in Figure S1 (Supporting Information), provides higher local detail. While the fine-grained view improves spatial resolution, it also potentially splits single spectral features into adjacent zones that are not independent. In our cohort, the model-based EMSC preprocessing standardizes the spectra as a whole, thus increasing the importance of ratios between intensities rather then their individual values. The importance of the intensity ratio for the model is most apparent for bands A (956–1030 cm^– 1^) and B (1030–1170 cm^– 1^) near Amide III (see Figure S1 in Supporting Information). The model learns this pattern and therefore expects a ratio change between A and B rather than independent increases or decreases. Accordingly, in the fine-grained analysis, B shows a positive contribution to the Sjögren prediction, whereas A shows a negative contribution. By contrast, the coarse analysis merges A and B into a single feature with an overall positive attribution (see Figure 5). These views are consistent rather than contradictory; opposite SHAP signs between neighboring zones primarily reflect the anticorrelation that is learned through preprocessing rather than conflicting biochemical effects. Therefore, zone-level attributions should be interpreted with caution. Consider preprocessing choices, the lack of independence among adjacent wavenumbers, and the model’s reliance on cohort-specific correlation structure.

Additionally, Figure 5 analysis confirms the relevance of the previously discussed spectral zones. Notably, the Amide III region (1210–1312 cm^–1^) shows a positive contribution (large red bar), reinforcing the role of altered protein-related bands in classification. The phosphate-related bands (1342 cm^–1^) and the COO^–^ stretching regions also contributed positively to the prediction.

Interestingly, the spectral region near 1312–1372 cm^–1^ corresponding to *CH*_3_ bending, had nearly no influence (light blue). While the spectral region 1477 to 1579 cm^–1^indicates a negative contribution, increasing the intensity of this zone strongly reduces the model’s confidence in predicting Sjögren’s disease. Recent Raman spectroscopy findings in serum from patients with Sjögren’s disease revealed decreased intensities of proline, tryptophan, and carotenoid-associated bands, suggesting systemic metabolic and compositional alterations.^36^ Correspondingly, ATR-FTIR spectra reflect these biochemical changes through variations in protein-related vibrational modes. The amide I (1600–1700 cm^– 1^) and amide II (1510–1580 cm^– 1^) regions arising from C=O stretching and N–H bending vibrations are particularly sensitive to alterations in protein secondary structure.^37^

The amide III region (1210–1312 cm^– 1^) and its adjacent zones B (1030–1170 cm^– 1^) and A (956–1030 cm^– 1^) also exhibit notable contributions. These bands originate primarily from C–N stretching and N–H bending in proteins, as well as C–O stretching in associated carbohydrate and nucleic acid moieties. The specific band around 1072 cm^– 1^, assigned to C–N stretching and O–H/C–O symmetric stretching, is linked to protein backbones (notably proline-rich sequences) and collagen-like structures, alongside possible carbohydrate and nucleic acid contributions.^38^ The decreased Raman signal of proline and tryptophan in SjD patients suggests disrupted protein synthesis and folding,^14^ which likely manifested in this study of ATR-FTIR as subtle changes in the amide I–III envelopes, particularly near 1072 and 1240 cm^– 1^, consistent with altered hydrogen bonding and backbone geometry. Collectively, these findings align with previous reports describing protein conformational and compositional changes in Sjögren’s disease. However, in ATR-FTIR analysis, we probe the total biochemical composition, so the observed IR bands represent collective vibrational responses from multiple biomolecules, including proteins, lipids, nucleic acids, and carbohydrates, rather than isolated molecular markers.

The global and individual-level SHAP analyses suggest that the model relies on spectral data patterns that coincide with known biochemical alterations observed in diseased states. Specifically, the model’s predictions appear to be influenced by phosphate and carbohydrate-associated spectral zone variations and increased intensities in regions corresponding to protein structural features, especially the amide bands. Figure 5 case-specific explanation reinforces this data-driven behavior. It shows how the model identifies spectral variations that distinguish Sjögren’s disease from the control group and reflects them through interpretable attributions.

### 3.3 Classification Model Results from ATR-FTIR Spectroscopic Analysis of Saliva

A variety of machine learning classification models were utilized to evaluate their performance. To perform independent validation, we applied stratified cross-validation at the patient level, meaning that all spectra from a given patient were assigned entirely to either the training or the validation set. In each fold, the network was randomly initialized, trained on the training subset, and evaluated on unseen data. Table 2 presents a summary of the classification performance for both the PCA-LDA and RamanNet models across various data set configurations. Results are displayed in terms of sensitivity and accuracy at both the spectral and patient levels.

**Table 2:**
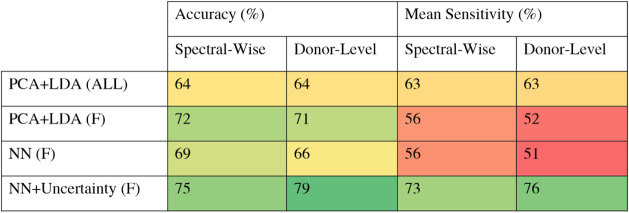
Classification Performance of PCA-LDA and ramannet Models across Different Data Conditions and Models was Evaluated under Different Dataset Conditions^a^

a The classical chemometric PCA-LDA model was evaluated on the full dataset, which included both male and female participants (“ALL”), as well as on a subset restricted to female donors (“F”). Neural Network (NN) Models were trained exclusively on female samples. although the deep learning approach alone did not outperform the PCA-LDA model, incorporating uncertainty-based data selection during training (nn+uncertainty), while keeping all spectra in validation, led to notable improvements in spectral- and donor-level accuracy and mean sensitivity (average sensitivity for control and Sjögren disease classes). Color shading is used to aid interpretation. dark green indicates better performance, and red indicates poorer performance.

The PCA-LDA model was first applied as a baseline method. When evaluated on the full data set (including both male and female participants), the model achieved moderate performance, with a spectral-level accuracy of 64% and a sensitivity of 63%. Given the known gender imbalance in the Sjögren’s class, a separate analysis was performed using only female samples to avoid influence of gender characteristics.^39^ Beyond this imbalance, sex-related bias theoretically could be mitigated by including a comparable number of male samples in the training process. However, previous studies in Raman signatures of saliva differ between male and female donors^40^ have shown that combining both sexes in a single model may reduce accuracy. Therefore, sex-specific diagnostic models are a more accurate alternative.

This gender-specific analysis showed improvements in spectral-level accuracy (72%) and patient-level accuracy (71%), but a decrease in spectral-level sensitivity (56%) and patient-level sensitivity (52%). Despite these moderate results, PCA-LDA serves primarily as a reference to assess the feasibility of spectral classification and to highlight the influence of demographic variability on model performance.

In contrast, the RamanNet neural network performed comparable to the PCA-LDA baseline during initial training, achieving similar accuracy and sensitivity. However, a significant decrease was observed at the patient level, with accuracy and sensitivity dropping to 66% and 51%, respectively. This decrease is likely due to label noise and the presence of ambiguous spectra within the Sjögren’s group. In addition, the relatively lower performance can be attributed to the higher parameter complexity of the neural network, which requires a larger amount of well-annotated data for effective learning compared to simpler models such as PCA-LDA.

Nevertheless, RamanNet has greater potential for scalability because its deep learning architecture can better adapt to larger and higher-quality data sets. To address the limitations caused by noisy labels and to improve model robustness, we incorporated Monte Carlo Dropout (MCD) for uncertainty-based sample selection in a second training phase, allowing the identification and exclusion of unreliable samples. Retraining the model with the complete set of control spectra and only the most confidently predicted Sjögren’s spectra significantly improved performance. The refined model, named NN+Uncertainty (F) in Table 2, achieved the highest accuracy (75%) and sensitivity (73%) at the spectral level, as well as improved patient-level metrics (accuracy: 79%, sensitivity: 76%).

These results show that PCA-LDA offers a strong and interpretable foundation for spectral classification, providing useful insights with low computational costs. While its performance was moderate in this study, optimizing parameters, such as the number of principal components, could enhance its effectiveness. In comparison, neural network models like RamanNet demonstrated greater scalability and adaptability, particularly when combined with uncertainty-aware techniques like Monte Carlo Dropout. As data sets expand and annotations improve, these models are well suited to capture complex, nonlinear patterns. Notably, our approach shows that incorporating uncertainty estimation can help mitigate the effects of mislabeled data, improve robustness, and support the development of reliable, scalable diagnostic tools.

### 3.4 Challenges and Practical Considerations

This proof-of-concept study demonstrates the feasibility of ATR-FTIR spectroscopy with machine learning for Sjögren’s disease using saliva collected through the Salimetrics passive drool method, which minimizes variability and is FDA-listed as an exempt collection device. Standardized saliva collection directly addresses one of the main barriers to reproducibility, ensuring that our spectral data are both reliable and clinically translatable. In parallel, advances in miniaturized ATR-FTIR platforms and lab-on-chip systems now provide portable and cost-effective devices capable of high diagnostic accuracy,^41,42^ while automated machine learning algorithms allow for real-time spectral analysis and automated classification with minimal operator input.^43^ Together, these developments make it realistic to position this technology first as a screening tool in outpatient or dental clinics, where a rapid, noninvasive saliva test could triage patients for further rheumatologic evaluation. As larger, multicenter validation studies are completed including comparison against overlapping conditions such as polypharmacy and head-and-neck radiation effects. we have planned these as future directions with the aim of FDA submission, ensuring that the technology can ultimately integrate seamlessly into existing clinical pathways.

Translating this approach from the laboratory to real-world screening requires addressing several practical considerations, such as automation, model adaptation, and clinical integration. Once the model is trained, preprocessing and inference can be performed almost instantaneously. However, standardized quality control procedures and adjusted protocols are essential for minimizing instrument- and site-specific variability. Unlike conventional biomarkers with fixed cutoff values, the deep learning model produces probabilistic outputs through its activation function (Sigmoid or SoftMax). When deployed across new instruments or patient populations, site-level validation will be necessary, as well as fine-tuning of the pretrained model if sufficient local data is available. This target adaptation helps preserve the model’s biochemical associations while ensuring clinical reliability.

To support both automated and human-in-the-loop review, interpretability outputs should accompany predictions. In practice, each spectrum could be reported with the following: (i) a calibrated probability, (ii) a zone-level SHAP plot highlighting relevant spectral bands, (iii) uncertainty and out-of-distribution checks, and (iv) a consistency flag derived from predefined attribution patterns. This would enable the identification of decisions driven by nonbiologically relevant features, such as baseline or instrument-related artifacts. When such cases are detected, or when uncertainty or out-of-distribution checks are triggered, the system could issue an alert recommending cautious interpretation, remeasurement, or collection of additional spectra before clinical decision-making.

## 4 Conclusions

This proof-of-concept underscores the emerging potential of integrating vibrational spectroscopy with uncertainty-aware machine learning for saliva-based diagnostics. Although still preliminary, the findings suggest that, with continued refinement and validation, this approach could evolve into a reliable early screening tool. Its noninvasive nature and adaptability make it particularly attractive for both conventional healthcare systems and resource-limited environments. To fully establish clinical utility, it will be essential to evaluate the technology in larger and more diverse patient cohorts, while also testing against potential confounding conditions such as polypharmacy and radiation therapy. Once sufficient statistical power is achieved and robustness is demonstrated, we anticipate pursuing FDA approval to enable broader clinical adoption.

ATR-FTIR is a perspective technique for medical diagnostic applications, but classic chemometrics might not always be sufficient for investigating samples where not all measured spectra are expected to have significant information for diagnostics. In such cases, uncertainty-aware AI models offer a flexible alternative that improves predictive performance compared with classical chemometric approaches and standard deep-learning models. Estimating uncertainty for such cases via ensemble models appears unsuitable for such studies due to a low data set sizes available for patient samples. In this study, to ensure unbiased evaluation of the result, the data analysis had to be even further restricted because Sjögren’s disease is more common in women.

Using uncertainty estimation, we manage to preselected the most reliable data for training, which then significantly improved the model performance on unseen data that did not undergo such preselection. The Monte Carlo Dropout approach made it possible to obtain an uncertainty estimation despite the very limited size of the data set. The designed routine can be further applied for similar tasks where a significant portion of spectra from the target class is expected to be indistinguishable from the control class.

This study highlights the potential for integrating vibrational spectroscopy with uncertainty-aware machine learning, which could significantly advance saliva-based diagnostic techniques. This integration has the potential to serve as a valuable tool for the early detection and monitoring of diseases in both traditional and resource-limited healthcare environments. It can play a crucial role in clinical translation by supporting automated preprocessing and inference, providing calibrated probability outputs, generating interpretable reports (such as zone-level SHAP and clinical-consistency flags), and improving robustness across instruments and sites.

Future work will include a direct comparative study of saliva ATR-FTIR and Raman spectroscopy using the same patient cohort to evaluate their relative performance. Additionally, we will test AI-based multimodal integration with both early feature fusion and late probability fusion to determine if combining these methods improves sensitivity, specificity, and overall reliability.

## 5 Highlights

•Application of ATR-FTIR and Neural Network for Sjogren disease diagnostics using saliva

•Improved prediction accuracy for classification via the Monte Carlo dropout approach for uncertainty estimation

•SHAP-based explainable AI revealed key spectral regions distinguishing Sjögren’s disease from controls.

## Supplementary Material



## Notes

Zone-level SHAP attribution for Sjögren’s disease detection using more refined zones (PDF)
