# Supplementary material for: ATR-FTIR Spectroscopy of Saliva and Machine Learning as a Screening Test for Sjögren Disease
Source: Anal Chem. 2025 Nov 18;97(47):26034–44. doi: 10.1021/acs.analchem.5c04238 (PMC12676517; doi:10.1021/acs.analchem.5c04238)
Supplement: Supplementary file 1 [file ac5c04238_si_002.pdf]

Supporting Information

ATR-FTIR spectroscopy of saliva and machine learning as a screening test for the Sjögren Disease

Jhonatan Contreras[1,2,#], *Bhavik Vyas*[3,#], *Oleg Ryabchykov*[1,2,*], *Melinda Larsen*[5], *Neil Gildener-Leapman*[4,5], *Thomas Bocklitz*[1,2], *Jürgen Popp*[1,2,3], *Igor K. Lednev*[3,5,*]

[1]Photonic Data Science Department, Leibniz Institute of Photonic Technology (IPHT), Albert-Einstein-Straße 9, 07745 Jena, Germany

[2]Institute of Physical Chemistry & Abbe Center of Photonics (ACP), Friedrich Schiller University Jena, Helmholtzweg 4, 07743 Jena, Germany

[3]Department of Chemistry and Center for Biophotonic Technology and Artificial Intelligence (CeBAI), University at Albany, SUNY, Albany, NY, 12222, USA,

[4]Division of Otolaryngology Head and Neck Surgery, Albany Medical College, Albany, NY, 12208, USA

[5]Department of Biological Sciences and The RNA Institute, University at Albany, SUNY, Albany, NY, 12222, USA

[#]Equal contribution

*Corresponding authors

Table of contents

Figure S1. Zone-level SHAP attribution using finer zones S2

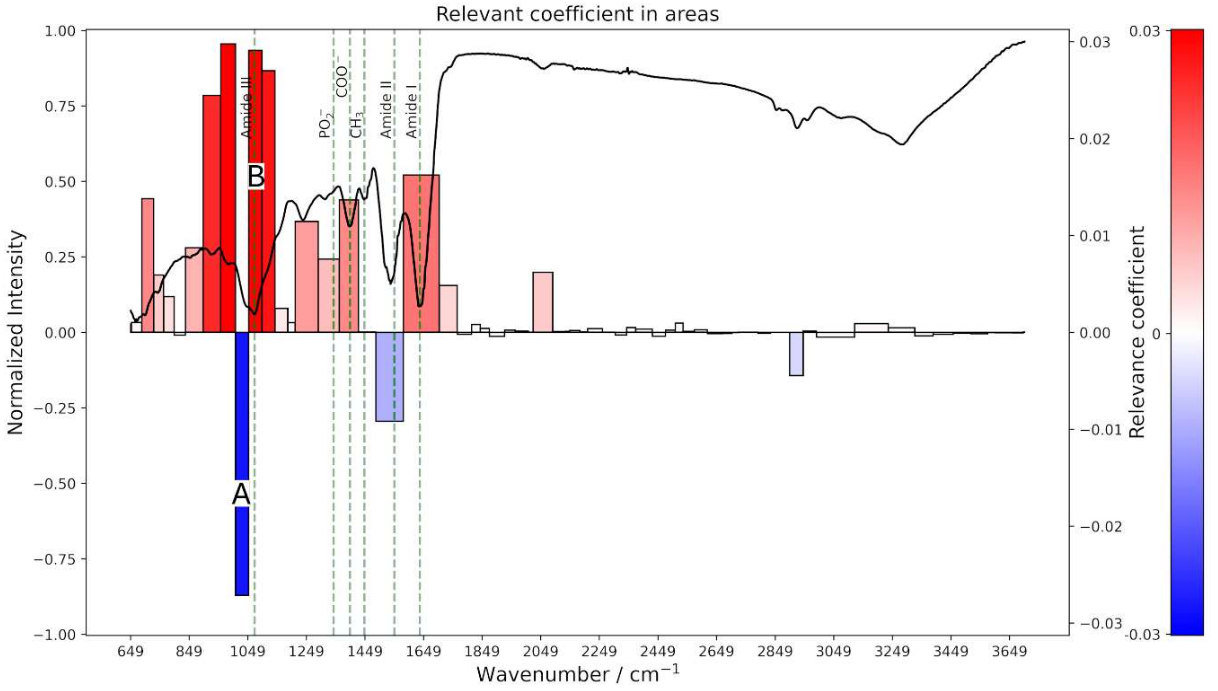

Figure S1. Zone-level SHAP attribution for an individual Sjögren spectrum using finer zones. Compared with Figure 5 (coarser zones), the smaller partitions provide higher resolution attributions but also pose interpretation challenges. Adjacent zones may capture correlated parts of the same spectral feature and thus show opposite SHAP signs. In the preprocessed data an inverse correlation between zones A (956–1030 cm^{-1}) and B (1030–1170 cm^{-1}, near Amide III) is observed, and the model therefore learns to expect the change in the ratio of A and B. Increases in B contribute positively to the disease prediction, whereas increases in A contribute negatively. On the other side, the merged AB region appears positive overall in coarse partitioning (Figure 5) when A and B are treated as a single joint feature. Consequently, zone-level attributions should be interpreted with caution, acknowledging dependencies introduced by preprocessing and learned by the model, and noting that adjacent wavenumbers are not independent variables.
